# The *Chlamydia* Effector TarP Mimics the Mammalian Leucine-Aspartic Acid Motif of Paxillin to Subvert the Focal Adhesion Kinase during Invasion*

**DOI:** 10.1074/jbc.M114.604876

**Published:** 2014-09-05

**Authors:** Tristan Thwaites, Ana T. Nogueira, Ivan Campeotto, Ana P. Silva, Scott S. Grieshaber, Rey A. Carabeo

**Affiliations:** From the ‡ Centre for Molecular Microbiology and Infection, Imperial College, London SW7 2AZ, United Kingdom,; § Bacteriology Section, Programme in Microbiology, Institute of Medical Sciences, School of Medical Sciences, University of Aberdeen, Aberdeen AB25 2ZD, United Kingdom, and; ¶ Department of Oral Biology, University of Florida School of Dentistry, Gainesville, Florida 32610

**Keywords:** Actin, Bacterial Pathogenesis, Cell Biology, Chlamydia, PTK2 Protein-tyrosine Kinase 2 (PTK2) (Focal Adhesion Kinase) (FAK), Signaling, Virulence Factor

## Abstract

Host cell signal transduction pathways are often targets of bacterial pathogens, especially during the process of invasion when robust actin remodeling is required. We demonstrate that the host cell focal adhesion kinase (FAK) was necessary for the invasion by the obligate intracellular pathogen *Chlamydia caviae*. Bacterial adhesion triggered the transient recruitment of FAK to the plasma membrane to mediate a Cdc42- and Arp2/3-dependent actin assembly. FAK recruitment was via binding to a domain within the virulence factor TarP that mimicked the LD2 motif of the FAK binding partner paxillin. Importantly, bacterial two-hybrid and quantitative imaging assays revealed a similar level of interaction between paxillin-LD2 and TarP-LD. The conserved leucine residues within the L(D/E)*X*LL*XX*L motif were essential to the recruitment of FAK, Cdc42, p34^Arc^, and actin to the plasma membrane. In the absence of FAK, TarP-LD-mediated F-actin assembly was reduced, highlighting the functional relevance of this interaction. Together, the data indicate that a prokaryotic version of the paxillin LD2 domain targets the FAK signaling pathway, with TarP representing the first example of an LD-containing Type III virulence effector.

## Teaser

**Background:** *Chlamydia* signals to the actin cytoskeleton via its virulence factor TarP during invasion.

**Results:** TarP contains a paxillin LD2-like motif that is recognized by the focal adhesion kinase (FAK). This interaction is required for signaling to the actin cytoskeleton.

**Conclusion:** *Chlamydia* TarP LD functions in FAK-dependent signaling to the actin cytoskeleton.

**Significance:** TarP is the first bacterial virulence factor that directly engages FAK.

## Introduction

Chlamydiae are obligate intracellular bacteria. They are auxotrophic for a number of essential nutrients and thus depend on the host cell for amino acids, nucleotides, and lipids among others (1). Their nutritional reliance on the host cell demands an efficient mechanism of invasion to gain access to the nutrient-rich intracellular environment. Upon entry, chlamydiae remain in vesicular compartments that are devoid of markers of the endolysosomal transport pathways (2) but exhibit interactions with nutrient-laden intracellular compartments, including the multivesicular bodies, lysosomes, endocytic recycling compartment, trans-Golgi network, the endoplasmic reticulum, lipid bodies, and peroxisomes (3–9). Chlamydiae differentiate from the metabolically quiescent elementary body to the vegetative reticulate body. This differentiation is concomitant with the loss of infectivity. At later time points, infectivity is regained through the conversion of the reticulate bodies to elementary bodies (10). This is followed by the release of infectious particles via host cell lysis or extrusion of intact inclusions (11).

The remodeling of the host cell actin cytoskeleton is usually required for efficient bacterial invasion. This is achieved either by the engagement of cell surface receptors by bacterial adhesin molecules or the translocation into the host cell cytosol of type III effectors that interact with host cell signaling machinery (12). For obligate intracellular pathogens like members of the family Chlamydiaceae, which have an absolute requirement for the protective intracellular niche for survival and replication, the efficient induction of actin cytoskeletal remodeling at the plasma membrane is vital (13, 14). Indeed, Chlamydiaceae have evolved to efficiently invade non-phagocytic epithelial cells utilizing the translocation of the type III effector TarP (15). This protein functions at the plasma membrane to mediate actin remodeling at the plasma membrane via host-signaling (16) and actin-nucleating activities (17), which cooperate to ensure efficient chlamydial uptake. More recently, *in vitro* experiments have revealed that TarP also binds filamentous actin (F-actin), with the activity narrowed to two distinct domains termed F-actin binding domain 1 (FAB1)^4^ and FAB2 (18). However, the direct actin nucleation or F-actin binding by TarP does not fully account for the observed actin assembly and bacterial uptake. Inactivation of critical signaling molecules like Rac, Cdc42, phosphatidylinositol 3-kinase, and the Arp2/3 complex also led to inhibition of invasion (14, 19–21), highlighting a prominent role of signal transduction in *Chlamydia*-mediated actin remodeling and invasion of non-phagocytic cells.

The focal adhesion kinase (FAK) signaling pathway is often the target by bacterial and viral pathogens and is triggered by the engagement of cell surface integrin molecules (22). This “outside-in” signaling results in the activation of FAK by autophosphorylation at tyrosine 397, leading to further phosphorylation by host cell kinases including Src (23, 24). With the exception of *Chlamydia pneumoniae* (25), integrin is thought to have no role in *Chlamydia* invasion (26).^5^ Two RNA interference screens have hinted at the involvement of FAK signaling in infection of epithelial cells by various chlamydial species (27, 28), but neither formally implicated it on the invasion process.

Signaling by FAK requires binding to focal adhesion-associated proteins via an array of protein-protein interaction domains within FAK, such as the N-terminal FERM domain, phosphorylated tyrosine and serine residues, and the C-terminal focal adhesion-targeting (FAT) domain (29). One protein that interacts with FAK is paxillin, and this binding requires the FAT domain of FAK and the LD motifs of paxillin, the latter having the signature sequence LD*X*LL*XX*L, with the leucine residues being conserved and essential (30, 31). This motif, of which paxillin has five, is predicted to fold into an amphipathic α-helix, forming a hydrophobic interface with the FAT domain (32). Of the five LD motifs of paxillin, the second (LD2) and fourth (LD4) motifs were implicated in binding FAK (33, 34).

Here we show that FAK recruitment at the sites of *Chlamydia caviae* invasion is mediated by the TarP protein. TarP deletion analysis identified a region at the conserved C-terminal half of the protein that harbors a putative LD-like motif. To date, LD motifs with FAK-related functions are only found in eukaryotes and to our knowledge have not been described for any bacterial Type III effectors. Interestingly, this motif is within the reported FAB1 of TarP. We verified the F-actin binding function of this domain in cultured cells but found this to be relevant only under conditions of overexpression in the cytosol. Plasma membrane-targeted TarP-LD exhibited only signaling-dependent actin assembly function.

Consistent with a signaling function, it was found that this chlamydial LD motif interacted directly with FAK to the same level as the LD2 motif of paxillin. They similarly recruited FAK, Cdc42, and actin to the plasma membrane, indicative of similar biochemical properties. Indeed, structural modeling indicated that like paxillin-LD2, TarP-LD could achieve an amphipathic α-helical conformation, forming a hydrophobic interface with the FAT domain of FAK. Genetic ablation of FAK abolished the ability of TarP-LD to recruit actin. Conversely, mutating the conserved leucine residues to alanine of TarP-LD abolished signaling to the actin cytoskeleton. Extending the *C. caviae* invasion studies of Subtil *et al.* (20), we determined that Cdc42 acts downstream of FAK and upstream of the Arp2/3 complex to mediate actin remodeling. Further supporting a biological relevance for the TarP-LD/FAK signaling, the recruitment kinetics of FAK and actin in the enteropathogenic *Escherichia coli* (EPEC) heterologous system was similarly transient as that observed in actual chlamydial infection. Altogether, the data indicate that in addition to F-actin binding, this region of TarP that contains the LD motif also functions in signaling, classifying TarP as the first Type III effector that mimics paxillin with regard to FAK binding.

## EXPERIMENTAL PROCEDURES

### 

#### 

##### Reagents, Cell Lines, and Organisms

Anti-HA.11 clone 16B12 mAb was purchased from Covance; anti-FAK (phospho-Tyr-397 (pY397-FAK)) pAb and anti-vinculin mAb were from Abcam, and anti-p34^Arc^ pAb was purchased from Millipore. Anti-rabbit or anti-mouse IgG secondary antibodies were either Alexa Fluor 488 or 594 were purchased from Invitrogen. Phalloidin conjugated to Alexa Fluor dye was purchased from Invitrogen. Cos7 (ATCC CRL-1651) and HeLa 229 (ATCC CCL-2.1) were routinely grown in DMEM supplemented with 10% FBS, 2 mm l-glutamine, and 10 μg/ml gentamicin. Subcultivation was at a 1:4 ratio. The cells were used at passage <15. FAK^−/−^ mouse embryo fibroblasts (CRL-2644) and matched FAK^+/+^ cells (CRL-2645), purchased from LGC standards, were cultured in DMEM + 10% FBS and subcultured at a 1:4 ratio. Cultured cells were grown in a humidified 5% CO_2_ incubator at 37 °C. *Chlamydia trachomatis* serovar L2 and *C. caviae* strain guinea pig inclusion conjunctivitis (GPIC) were propagated in HeLa cells grown in DMEM + 10% FBS supplemented with gentamicin (10 μg/ml). Harvest of elementary bodies was by discontinuous density gradient centrifugation in Renografin (Bracco Diagnostics), as previously described (35).

##### Cloning

A summary of all primers used and plasmids generated in this study is provided in Tables 1. The LD (*LEHLLPQL;* Ser^640^–Pro^740^), mutLD (*A**EH**AA**PQI*; Ser^640^–Pro^740^), and GPIC^714–880^ fragments were PCR-amplified from *C. caviae* GPIC TarP clone (36) and paxillin LD2 (*LDRLLLEL;* Lys^125^–Glu^186^) from pEGFP-N3-paxillin (Ref. 37; obtained from Prof. Rick Horwitz (University of Virginia) through Addgene) using primer pairs 3–4, 5–6, 7–8, and 15–16, respectively. The TarP deletion variants (Met^1^–Thr^714^ and Met^1^–Thr^639^) were generated from GPIC TarP clone (36) using primer pairs 9–10 and 11–12 engineered with Kpn1 linkers for fusion with the TirM derivative of the TirMC plasmid (pKC87). This plasmid has been previously described by Campellone *et al.* (37) and was generously provided by Prof. John Leong (Tufts University). Construction of TirM involved PCR amplification of part of TirMC that contained amino acids 1–66, which encodes the essential membrane targeting domain of the Newcastle Disease Virus HN surface protein, the hemagglutinin (HA) epitope tag, and Tir amino acids 260–395. The C region of TirMC was removed, and in its place a unique Kpn1 site was introduced at the 3′ end of the TirM open-reading frame using primer pair 1–2. This facilitated the cloning of TarP fragments to create TirM-TarP fusion derivatives. Blunt-end TirM PCR products were cloned directionally by TOPO® cloning into a pENTR™/D-TOPO® entry vector (Invitrogen) to create pENTR-TirM. The products generated for TarP-FL, its deletion variants, and isolated regions of interest were digested with KpnI and subcloned into linearized pENTR-TirM to generate translational fusions with TirM at the N terminus. Expression clones were generated through LR recombination between entry clone and pcDNA-Dest40 (Invitrogen), which had the CMV promoter to drive expression in mammalian cells. For coexpression in the bacterial two-hybrid system, PCR fragments were generated to include flanking KpnI sites and were cloned into the same sites in pUT18C and/or pKT25 (kind gifts from Prof. Alain Filloux, Imperial College). All constructs were verified by DNA sequencing.

**TABLE 1:** **List of primers** Fwd, forward; Ref, reverse.

Primer ID	Primer name	Sequence (5′-3′)
1	TirM Fwd	CACCATGGGCTTAGGAAATGATGAAAGGGAACGG
2	TirM Rev	AGTATTGGTACCCCTGTTCTGCCGGCTG
3	LD Fwd	GTAAATAGGTACCGTATGTCTTCTGAATCACGAGCC
4	LD Rev	GTCGCTTGTGGTACCTTATTTATCTCCCCCTGTACC
5	mutLD Fwd	AAAATAATAGGTACCGTATGAGTGCTGCAGGTGGTGA-
		GGGCGCAGAAGGAGCCGAGCATGCAGCACCACAGGCA
6	mutLD Rev	GGCGCCGCGGGTACCTTAAGGAGTCGTTCTTTCTGC
7	TarP^714–880^ Fwd	GAGGGCAGGTACCGTATGACTAGCAGTTCTGCA
8	TarP^714–880^ Rev	ATAGTGGATGGTACCTTAGGAGTGTCTTTGAGG
9	TarP^1–639^ Fwd	GCCCTCAGGTACCGTATGACTAGTCCTATT
10	TarP^1–639^ Rev	GCAGCTAGGTACCGTATGAAGCCTACAGTATTGTTA
11	TarP^1–714^ Fwd	GCCCTCAGGTACCGTATGACTAGTCCTATT
12	TarP^1–714^ Rev	GTCGCTTGTGGTACCTTATTTATCTCCCCCTGTACC
15	Pax LD2 Fwd	CCAGTAAAGGTACCGTATGAAGTCTGCAGAACCA
16	Pax LD2 Rev	TTGCGAGGGTACCTTACTCTGGGATGACATA

##### Transfection and Indirect Confocal Microscopy

Cos7 cells were either co-transfected with 100 ng of full-length TarP (TarP-FL) and pEGFP-FAK or transfected with only TarP-FL or the TarP-LD domain using Lipofectamine 2000 (Invitrogen) as described by the manufacturer. Similar procedures were followed in the Cdc42 studies using GFP-Cdc42 constructs generously provided by Prof. Michael Way (Cancer Research UK). At the required time post-transfection, the cells were fixed by adding 4% paraformaldehyde. Antibodies diluted in 1× PBS to their respective working concentrations (anti-HA.11 clone 16B12 1:500, anti-pFAK (phospho-Tyr-397) 1:750 and anti-vinculin 1:250 were added to fixed cells and incubated 37 °C for 1 h. Actin was detected with phalloidin (1:250 dilution) conjugated to Alexa Fluor dye. Coverslips were mounted onto glass slides using Mowiol. Sample visualization was performed at room temperature on a laser scanning microscope (LSM 510; Carl Zeiss) using an oil immersion PlanApochromat 63x/1.40 NA differential interference contrast. Images were processed using NIH ImageJ freeware or Adode Photoshop CS5. Pixel intensities were measured using ImageJ freeware. Incidences of colocalization were assessed visually in single-blind experiments by someone not involved with the image acquisition.

##### Oligopeptide Pulldown and Immunodetection

Proteins from infected HeLa cell lysates and precipitates from oligopeptide pulldown were monitored by Western blot, probed with a monoclonal anti-pFAK (phospho-Tyr-397) or anti-FAK antibody, respectively, and visualized by chemiluminescence. HeLa cell lysates were prepared as described previously (14) with the following modifications. Infections of cells exposed to serovar GPIC EBs (multiplicity of infection = 50) were allowed to proceed for 10, 30, 60, 90, or 120 min. Oligopeptide pulldown was as performed as follows. Custom-synthesized oligopeptides with sequences EGAEG*LEHLLPQI*RSHLDDAFDQQGN (WT; LD motif in italics) and EGAEG*A**EH**AA**PQI*RSHLDDAFDQQGN (Mutant: substituted residues underlined) purchased from Sigma-Genosys were designed to include an N-terminal biotin residue. Prebinding of the oligopeptides to the streptavidin-coupled Dynabeads (Invitrogen) was from the manufacturer's instructions. Briefly, 100 μl of bead slurry was incubated with a 10-fold excess (200 pmol) of oligopeptides at 4 °C for 1 h, and excess oligopeptides were removed by magnetic separation of the beads followed by 5 washes with the radioimmune precipitation assay buffer (16). Post-nuclear supernatants were prepared as previously described (14) and added to the oligopeptide-coated beads. Binding was allowed to proceed at 4 °C for 1 h. The beads were removed by magnetic separation and washed 3 times with radioimmune precipitation assay buffer. 150 μl of 2× Laemmli buffer was added to elute bound proteins. The samples were boiled before SDS-polyacrylamide gel electrophoresis.

##### Bacterial Infection

Δ*tir* and Δ*escN* EPEC mutants are deficient for Tir and the critical ATPase that powers the type III secretion system, respectively. Both mutants behaved similarly in adhesion, recruitment, and signaling assays, and results for Δ*tir* are presented here. For quantification purposes, data for the Δ*tir* mutant were presented. The Δ*escN* mutant produced clusters that hindered accurate distinction and enumeration of individual bacterium. For Δ*tir* or Δ*escN* EPEC, bacterial cultures were primed for infection as previously described (38) with the following modification. A 1:500 dilution of overnight culture was used to prime the bacterial culture for infection. Cos7 and FAK^−/−^ cells were grown in 24-well cell culture plates to 80% confluence. The cells were transfected with 100 ng of plasmid DNA. Transfected cells were incubated at 37 °C in a humidified incubator for 32 h before infection. Cells were infected as previously described (38). The infection was allowed to proceed for 4 h in the presence of gentamicin (200 μg/ml; Invitrogen) after the first hour. Δ*tir* or Δ*escN* EPEC were visualized using DAPI (1:1000). For experiments investigating the Arp2/3 requirement, the Arp2/3 complex inhibitor I (CK-666; Millipore) or the negative control CK-689 (Millipore) were added at 100 μm 1 h before infection and remained for the duration of infection. p34^Arc^ recruitment was monitored using an anti-p34^Arc^ 1:300. For infection with *Chlamydia*, HeLa, FAK^−/−^, and FAK^+/+^ cells were infected as previously described (19) with the following modifications. Cells were infected at a multiplicity of infection of 50 for *C. caviae* GPIC and centrifuged (1500 rpm for 3 min) at 4 °C to allow maximum chlamydial adherence to target cells (20).

##### Invasion Assay

Efficiency of *C. caviae* strain GPIC invasion was performed using a double staining and fixation protocol as described previously (13, 20) with the following modifications. FAK^−/−^, FAK^+/+^, or HeLa cells were infected *C. caviae* at multiplicity of infection of 1 and centrifuged at 1500 rpm for 3 min at room temperature to synchronize infection. For experiments investigating the Arp2/3 requirement, CK-666 or CK-689 were added at 100 μm 1 h before infection and kept for the duration of infection. Unattached EBs were removed by washing cells 3 times at 4 °C with Iscove's modified Dulbecco's medium. Prewarmed Iscove's modified Dulbecco's medium was added, and the infection was allowed to proceed at 37 °C for the indicated time post-infection (p.i.). Coverslips were washed 3 times in PBS and fixed with 4% paraformaldehyde for 15 min. Extracellular EBs were labeled with a mouse IgG anti-*C. trachomatis* LPS 1:750 (Abcam) primary and goat anti-mouse IgG Alexa Fluor 488-conjugated secondary antibody (Invitrogen). Cells were permeabilized with 0.25% Triton X-100 for 5 min and washed 3× with PBS before a secondary fixation step with 4% paraformaldehyde for 15 min. Total EBs were labeled with an rabbit anti-*Chlamydia* antibody as the primary antibody at 1:750 dilution (Abcam) and a goat anti-rabbit IgG Alexa Fluor 594-conjugated secondary antibody (Invitrogen).

##### Live-cell Microscopy

The pEGFP-C1-FAK and EGFP-Cdc42 plasmids previously described (39, 40) were generously provided by Dr. J. Thomas Parson (University of Virginia) and Dr. Michael Way (Cancer Research UK), respectively. Cos7 cells were subcultured onto 25-mm glass coverslips in 6-well plates. For *Chlamydia* infection, cells were transfected with the pEGFP-C1-FAK using the FuGENE6 transfection reagent (Roche Applied Science) per the manufacturer's instructions. The coverslips were placed in a coverslip holder for microscopy, and temperature was maintained at 37 °C throughout the experiment using an objective heater (Bioptechs, Butler, PA). At 18 h post-transfection, cells were infected with CellTracker^TM^ Red 4-({[4-(chloromethyl)phenyl]carbonyl}amino)-2-(1,2,2,4,8,10,10,11-octamethyl-10,11-dihydro-2H-pyrano[3,2-g:5,6-g']diquinolin-1-ium-6-yl)benzoate (CMTPX)-labeled *C. caviae* serovar GPIC EBs. For experiments involving infections with Δ*tir* EPEC, Cos7 cells were co-transfected with GFP-FAK and TarP-FL, TarP-LD, Pax-LD2, or TirM for 32 h. During Δ*tir* EPEC priming (see above for details), the DNA dye DRAQ5 (Abcam) was added at a 1:1000 dilution to label the particles. Infected cells were monitored after 1 h of infection in the presence of gentamicin (200 μg/ml; Invitrogen). Images were collected live with a PerkinElmer Life Sciences UltraView Spinning Disc Confocal system connected to a Nikon Eclipse TE2000-S microscope using a 60×, 1.4 NA oil immersion objective. Recruitment of EGFP-C1 FAK or EGFP-Cdc42 was observed at 30-s intervals.

##### Bacterial Two-hybrid

The pKT25 vector containing the FAK FAT domain and pUT18C vector harboring TarP-LD, TarP-mutLD, or Pax-LD2 were co-transformed into the adenylate cyclase-deficient *E. coli* strain DHM1. This was followed by a Miller assay as described (41).

##### Structural Modeling

Sequence alignment between the L2 LD, GPIC LD, and LD2 paxillin domains was performed using the program T-Coffee (42). The sequence alignment and the structure of the LD2 paxillin domain were used as input for the comparative homology-modeling program MODELLER (43). The resulting L2 LD and GPIC LD models were superposed to the structure of the LD2 paxillin domain bound to the FAT domain of FAK with the graphical program PYMOL 1.4. Validation of the geometry of the models was performed with MOLPROBITY (44), and the surface electrostatic potential representation was generated with PYMOL 1.4.

##### Statistical Analysis

All statistical analyses were run using the program StatPlus. Unless stated otherwise, statistical differences were tested using one-way analysis of variance (ANOVA) and Tukey-Kramer post hoc test. Spearmann's rank order correlation test was used to determine the strength of association between the recruitment kinetics of phospho-active FAK, pEGFP-C1-FAK, or both by *C. caviae* GPIC. Box and whisker plots show range and the lower and upper quartile values (*box*) with medians (*horizontal line*) and means (*diamond*). Whiskers extend from each end of the box to the 10th and 90th percentiles. Where stated, the significance level α was set to 0.05 or 0.01. All *p* values ≤ α were considered statistically significant. *q* values were provided for comparison to the *q*_critical_ for α of 0.05 or 0.01.

## RESULTS

### 

#### 

##### FAK Localizes to Sites of Chlamydial Attachment, Is Phosphorylated Early in Infection, and Is Required for Invasion

**FIGURE 1.**
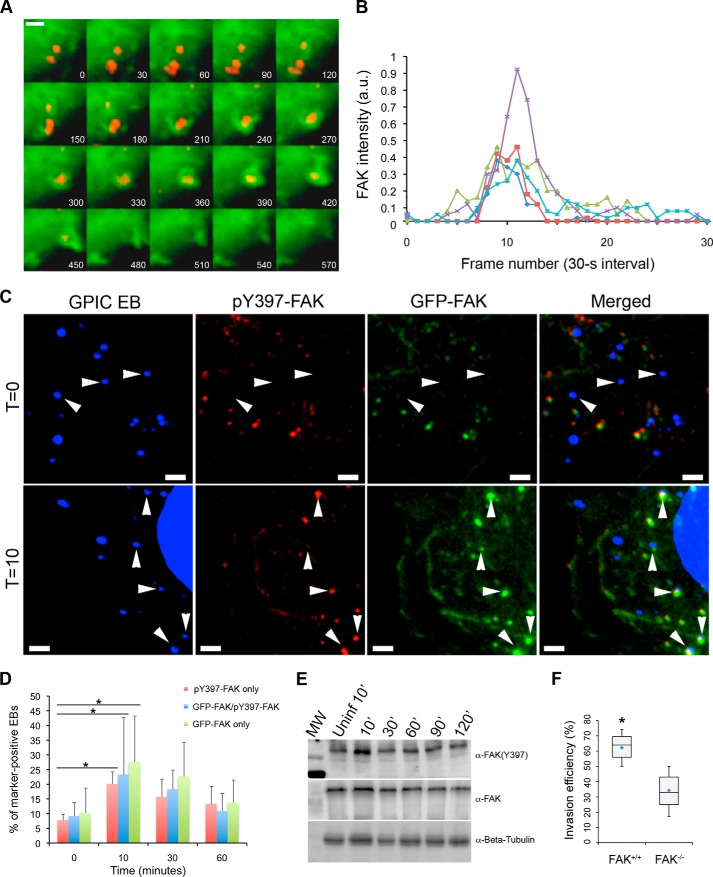
**FAK transiently localizes to sites of chlamydial adherence and is required for invasion.** *A*, binding of CMTPX-labeled *C. caviae* strain GPIC (*red*) to cells induces a highly localized and transient recruitment of EGFP-FAK (*green*). The images were obtained by live cell microscopy at 30-s intervals. *Scale bar*, 5 μm. *B*, pixel intensities for representative areas of EGFP-FAK recruitment were enumerated and plotted as pixel intensity in arbitrary units (*a.u.*) over time. *C*, Cos7 cells were infected with *C. caviae* (GPIC) EBs (DAPI, *blue*) and stained with an anti-phospho-Y397 FAK (*pY397-FAK*; *red*) antibody. *White arrowheads* indicate pY397-FAK and GFP-FAK colocalizing with GPIC EBs. *Scale bar*, 5 μm. *D*, EB colocalization with phospho-FAK (*pY397-FAK*), GFP-FAK, or both was enumerated, and data expressed are expressed as as the percentage of total EBs displaying colocalization. The *asterisk* and *bars* indicate significant difference between pY397-FAK, GFP-FAK, or both from *T* = 0 to *T* = 10 (one way ANOVA, Tukey's post hoc test q = 4.7, α = 0.01, pY397-FAK *p* < 0.005; GFP-FAK *p* < 0.004; both *p* < 0.03). Correlation of the recruitment kinetics was tested using Spearmann's test for correlation (one-tailed; *p* < 0.005). Data from three independent experiments were represented as the mean ± S.D. *E*, infection-dependent induction of FAK phosphorylation (Tyr-397). Solubilized HeLa lysates were probed with an anti-phospho-Tyr-397 FAK or anti-FAK antibody to monitor FAK activation or total FAK, respectively. The lane labeled (*Uninf 10′*) refers to a parallel sample collected at the 10-min time point after mock-infection. β-Tubulin served as a loading control. *F*, *C. caviae* GPIC invasion required FAK. Infection of FAK^+/+^ and FAK^−/−^ cells was allowed to proceed up to a 10-min post-temperature shift. Data are from a minimum of 100 cells from three independent experiments. The *asterisk* indicates statistical significance (ANOVA *p* < 0.005. and post hoc testing Tukey-Kramer; α = 0.01, q = 4.7).

To gain insight into the involvement of FAK in chlamydial invasion, its localization during infection was investigated by confocal microscopy of live and fixed samples. Using live-cell imaging, we observed the immediate and specific localization of EGFP-FAK to the *Chlamydia* entry sites (Fig. 1*A*). EGFP-FAK fluorescence intensity at areas of EB attachment was quantified and adjusted relative to the neighboring regions lacking EBs. The data indicated that the EB-localized fluorescence only persisted for an average of 180 ± 60 s (Fig. 1*B*). This rapid and transient recruitment was consistent with previously published data on the recruitment of Rac GTPase, the Arp3 subunit of the Arp2/3 complex, and actin (13, 14).

We then investigated if endogenous phosphoactivated FAK (pY397-FAK) localized to the sites of entry as recognized by an anti-phospho-FAK (pFAK) antibody. Time-course analyses of recruitment of EGFP-FAK and/or endogenous pY397-FAK were performed, and the recruitment profiles were compared. The respective recruitments of EGFP-FAK and pY397-FAK to adhered EBs in fixed samples exhibited similar kinetics, with an increase at 10 min p.i., before steadily decreasing between 30 and 60 min p.i. (Fig. 1, *C* and *D*). Statistical analysis revealed that the increases in recruitment from *t* = 0 to *t* = 10 were significant (*p* < 0.005, ANOVA and Tukey-Kramer post hoc test). Furthermore, there was a high degree of temporal correlation between the two recruitment profiles (*p* < 0.005, Spearmann's test for correlation) (Fig. 1*D*), suggesting that the two events were related. When the levels of pY397-FAK was monitored in whole cell lysates collected from infected cells at different time points, a peak at 10 min p.i. was observed followed by a steady decrease at 30 and 60 min p.i (Fig. 1*E*), a result that paralleled the microscopy data. Importantly, the increase in the levels of pY397-FAK from uninfected to *t* = 10 suggested that phosphorylation was infection-dependent. Together, the data indicate that FAK recruitment and its phosphorylation at Tyr-397 are early and transient events during infection.

Finally, the role of FAK in chlamydial invasion was evaluated using FAK^+/+^ and FAK^−/−^ mouse embryo fibroblasts (MEFs). Chlamydia adherence to and invasion of both cell types were synchronized by centrifugation and incubation with prewarmed media, respectively. Infections were stopped at the specified time points by paraformaldehyde fixation. The samples were processed for immunofluorescence to identify and quantify uninternalized and total chlamydial EBs as described under “Experimental Procedures.” A statistically significantly different efficiency (*p* < 0.005, ANOVA and Tukey-Kramer post hoc test) in invasion between wild type and FAK^−/−^ fibroblasts was readily observable at 10 min p.i. (Fig. 1*F*). The reduced invasion efficiency observed in FAK^−/−^ MEFs was consistent with the results obtained from two independent RNA interference screens (27, 28). Collectively, the highly localized recruitment of FAK, its early induction of phosphoactivation, and the reduction of invasion efficiency in FAK^−/−^ cells all point to an essential role of FAK in *Chlamydia* invasion.

##### Ectopically Expressed LD Motif Colocalized with pY397-FAK and Actin at Stress Fibers

During *Chlamydia* invasion, the recruitment of some of the host signaling molecules to the sites of adhesion could be attributed to TarP. For example, in *C. trachomatis*, the recruitment of Sos1 and Vav2 guanine nucleotide exchange factors (GEFs) and the p85 subunit of the phosphatidylinositol 3-kinase was dependent on specific phosphotyrosine residues found in the N-terminal half of TarP (16). Thus, there is precedent for TarP acting as a molecular scaffold to which signaling molecules can be recruited.

**FIGURE 2.**
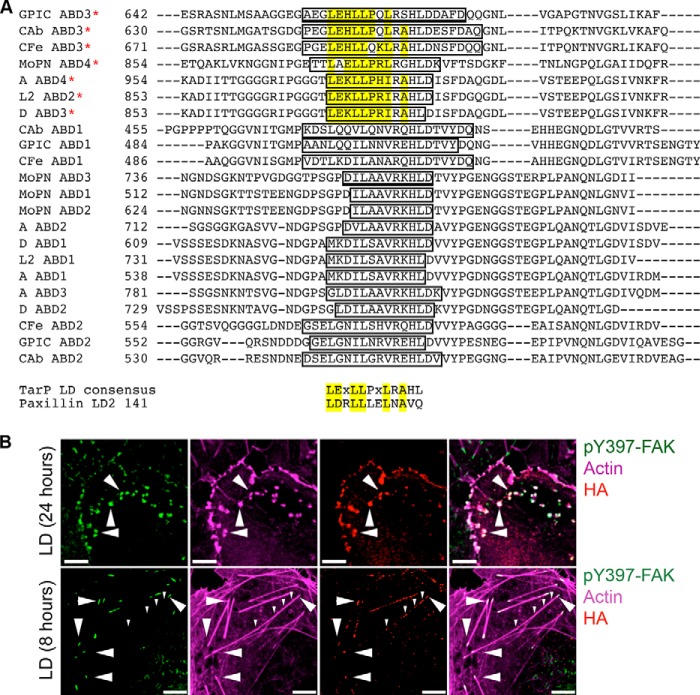
**Focal adhesion components associate with TarP and its conserved LD-like motif.** *A*, ClustalW sequence alignment showing the putative actin binding domains (*ABD*; *open box*) from Tarp orthologs with the conserved residues of the LD motif highlighted in *yellow*. The *red asterisk* indicates the recently identified F-actin binding domains (FAB1). The consensus sequence of the LD domain within TarP was aligned with the paxillin LD domain to highlight homology. Species analyzed include *C. caviae* (GPIC), *Chlamydophila abortus* (*CAb*), *Chlamydophila felis* (*CFe*), *Chlamydia muridarum* (*MoPn*), *C. trachomatis* serovars A, L2, and D. *B*, the TarP-LD-like motif was ectopically expressed in Cos7 cells for either 24 or 8 h and tested for the ability to co-recruit pY397-FAK and actin (*white arrowheads*). When transient expression was limited to 8 h rather than 24 h, the TarP-LD specifically localized to FAK-rich focal adhesion structures and to various points along actin stress fibers. *Large arrowheads* in the 24-h panel indicate the co-recruitment of pY397-FAK and actin to TarP-LD aggregates, whereas in the 8-h panel, they indicate the localization of TarP-LD to focal adhesion structures. *Small arrowheads* indicate the localization of TarP-LD along the F-actin. *Scale bars*, 10 μm.

The interaction of FAK with a variety of binding partners either depends on phosphorylation (on tyrosine, serine, and/or threonine) or on signaling domains (FERM and FAT) (45). Amino acid sequence analysis of TarP orthologues from various chlamydial species for mammalian-like signaling motifs identified a domain (LD) similar to paxillin and thus potentially could be recognized by the FAT domain of FAK (Fig. 2*A*). These highly conserved LD-like motifs overlapped a region originally characterized as non-functional globular actin binding domain. In addition to the amino acid sequence conservation, the positions along the TarP orthologs were also conserved, with only the most C-terminal of the actin binding domains harboring the LD-like motifs. Thus, there appears to be both sequence and position conservation among the TarP-LD motifs found in various TarP orthologues. The most distinct feature of the TarP-LD sequences (LE*X*LLP*X*LRAHL) was the conserved leucine residues, which in paxillin LD2 are critical for FAK binding. The putative signaling function would be in contrast to the F-actin binding activity previously assigned to this region of TarP (18). Therefore, it was necessary to address this potential discrepancy. Using a similar approach used by the Jiwani *et al.* (18), ectopically expressed TarP-LD colocalized with F-actin as previously reported. However, when the duration of expression was limited to 8 h, colocalization was predominantly at the tips of stress fibers, which coincidentally were also positive for pY397-FAK (Fig. 2*B*). Overall, the data raised the possibility that the FAB1 motif may have an alternate function, which is to mediate TarP/FAK interaction at the plasma membrane during invasion.

##### The Highly Localized and Transient Recruitment of FAK to the Plasma Membrane Depended on TarP

**FIGURE 3.**
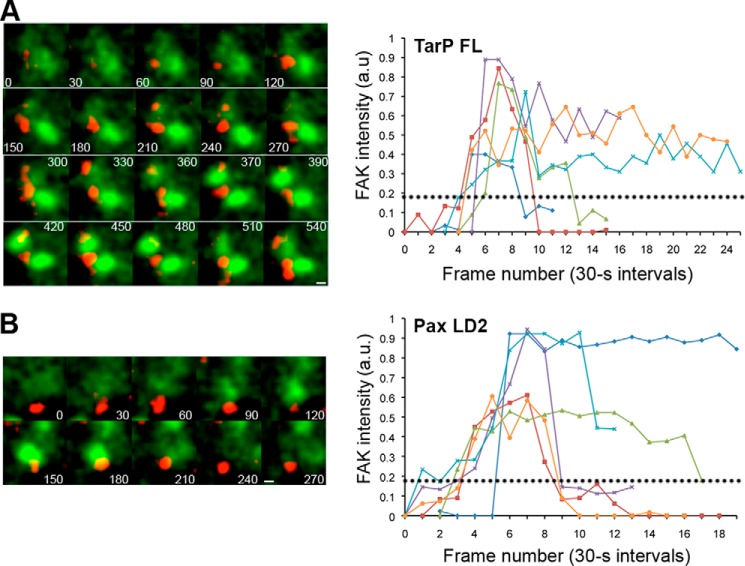
**Plasma membrane-localized TarP functions to recruit FAK.** *A* and *B*, DRAQ5-labeled Δ*tir* EPEC (*red*) bound to cells co-expressing full-length TarP (TirM-TarP-FL; *A*) or TirM-Pax-LD2 (*B*) with pEGFP-FAK (*green*) induced a highly localized and transient recruitment event. The images were obtained by live cell microscopy at 30-s intervals. Pixel intensities for representative areas of EGFP-FAK recruitment in TarP-FL- or Pax-LD2-expressing cells were enumerated and plotted as pixel intensity in arbitrary units (*a.u.*) over time. Background fluorescence intensity for neighboring regions was obtained to adjust pixel intensities at the regions of interest. The *dotted line* represents the average intensity for EGFP-FAK recruitment by Δ*tir* EPEC in cells expressing the negative control, TirM. *Scale bar*, 5 μm.

**FIGURE 4.**
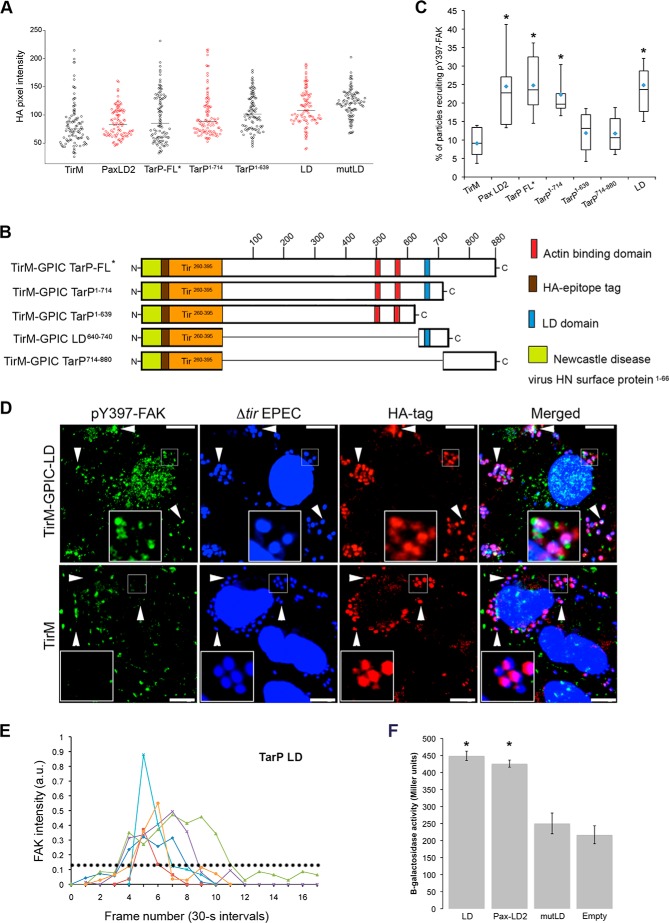
**TarP-mediated FAK recruitment is direct and requires the LD domain.** *A*, the clustering ability of the various constructs upon infection by EPEC was evaluated by quantifying pixel intensity and found to be similar. The plasma membrane-clustered labels were specifically monitored because they were the functional subpopulation with regard to signaling and recruitment of FAK and actin. *B*, diagram of the various TirM-TarP fusion constructs used to identify the region in TarP required for FAK recruitment. The numbers indicate amino acid positions encoded within the *C. caviae* TarP gene. * denotes full-length TarP. *C*, adhered EPEC able to recruit phospho-FAK were enumerated for the constructs indicated, and data are represented as a *box and whisker plot*. Approximately 500–750 particles were counted for each construct. The *asterisks* indicate significance relative to TirM control (ANOVA; *p* < 0.005 and post hoc testing Tukey-Kramer; α = 0.01, q = 5.11). *D*, representative images analyzed to arrive at the data shown in *C*. Cos7 cells transfected with plasmids encoding progressive TirM-TarP-LD and TirM negative control were infected with Δ*tir* EPEC to induce clustering of the fusion protein. The *white arrowheads* indicate colocalization phospho-active FAK (pY397-FAK; *green*) with Δ*tir* EPEC (*blue*). Anti-FAK (phospho-Tyr-397) and anti-HA antibodies were used to visualize pY397-FAK (*green*) or TarP^1–714^/TarP^1–639^/TarP-LD (*red*), respectively. *Insets* show a magnification of a selected area of the cell. *Scale bars*, 10 μm. *E*, the kinetics of EGFP-FAK recruitment was found to be rapid and transient. Pixel intensities for representative areas of EGFP-FAK recruitment to Δ*tir* EPEC in TarP-LD-expressing cells were enumerated from live cells and plotted as pixel intensity in arbitrary units (*a.u.*) over time. Background fluorescence intensity for neighboring regions was used to normalize the data. *Scale bar*, 10 μm. *E*, β-galactosidase activity of *E. coli* DHM1 strains co-expressing Cya25-FAT and Cya18-TarP-LD, Cya18-Pax-LD2, Cya18-TarP-mutLD, or Cya18-Empty. Activity is expressed as Miller units. ANOVA; *p* < 0.01 and post hoc testing Tukey-Kramer; α = 0.01, q = 4.91).

Mutagenesis of a putative invasion factor of an obligate intracellular pathogen would make impossible the isolation of such mutants. Therefore, we utilized an EPEC-based heterologous system (38) that enabled the expression and plasma membrane targeting of various TarP mutants for downstream functional analyses. In brief, TarP (or various deletion derivatives in subsequent experiments) was fused with a truncated version of the translocated intimin receptor (Tir) from EPEC. The truncated version of Tir used in this assay lacked any original signaling components while maintaining the extracellular intimin binding domain to allow adherence of Δ*tir* or Δ*escN* EPEC to the host cell. A viral membrane-targeting sequence replaced the NH_2_ terminus of Tir to promote sufficient expression of the fusion protein at the plasma membrane. This was followed by an HA tag for immunofluorescence detection. By fusing this truncated Tir to TarP or various TarP deletion derivatives, we could ascertain the signaling and actin assembly functions of individual motifs of TarP. In this assay cells expressing full-length TarP (TirM-TarP-FL) (Fig. 3*A*) were infected with Δ*tir* EPEC and monitored for EGFP-FAK recruitment under live cell conditions. As a positive control, the LD2 motif of paxillin, the FAK *in vivo* binding partner, was also investigated (Fig. 3*B*). From the series of images, paxillin LD2 was capable of recruiting FAK. Monitoring the recruitment of EGFP-FAK by live-cell imaging revealed transient recruitment kinetics for both TarP-FL and paxillin LD2. Quantification of EGFP-FAK fluorescence intensities revealed a recruitment duration of ∼180s and with the peak occurring 90s from the time of EGFP-FAK appearance. In the majority of cases (>85%), FAK recruitment in TarP-FL- or LD2-expressing cells was found to be transient following a similar profile to that observed during *Chlamydia* infection (Fig. 1*A*). The data indicated that TarP was sufficient to recruit FAK to the plasma membrane. In all cases the HA signal intensities associated with the clustered TirM-TarP-FL and TirM-LD2 were found to be similar (Fig. 4*A*), ensuring that any observed differences in pY397-FAK recruitment were not due to variations in the expression levels.

##### FAK Recruitment by TarP Requires the LD Domain

Having established that TarP was sufficient to recruit FAK and a putative region (LD) was identified, various derivatives of TarP deleted at the region harboring the LD motif were constructed (Fig. 4*B*). By analyzing the various deletion derivatives, the FAK-binding role of the putative LD motif in Tarp could be confirmed. Furthermore, this approach would enable the identification of regions outside the LD motif that could interact with FAK. As above, Cos7 cells were transfected with the various constructs infected with Δ*tir* or Δ*escN* EPEC, and FAK recruitment was monitored. Data for or Δ*tir* EPEC are shown. First, the levels of recruitment of the TirM-TarP deletion derivatives to the plasma membrane were quantified by immunofluorescence microscopy of anti-HA-stained samples and found to be similar (Fig. 4*A*). Untransfected cells were devoid of adhered Δ*tir* EPEC and HA staining at the plasma membrane. We observed that the full-length construct and TarP^1–714^ retained the ability to recruit pY397-FAK, whereas deletion further into the protein, as in TarP^1–639^, led to the loss of this recruitment. Therefore, the region between residues 639 and 714 mediated FAK recruitment (Fig. 4*C*). Indeed, TarP^640–740^ (herein termed as TarP-LD) was sufficient in recruiting pY397-FAK to the plasma membrane. Scoring individual EPEC particles for colocalization with pY397-FAK showed an increase for TarP-FL (2.7-fold), TarP^1–714^ (2.5-fold), TarP-LD (2.4-fold), and paxillin LD2 (2.4-fold) relative to the TirM-only negative control (Fig. 4*C*; *p* < 0.005; ANOVA and Tukey Kramer post hoc test). Interestingly, TarP-LD yielded a similar recruitment frequency value as paxillin LD2. Representative images from which the data were derived are shown in Fig. 4*D*.

To examine the kinetics of FAK recruitment by TarP-LD, we conducted a series of short-term (<10 min) live-cell imaging experiment using cells expressing pEGFP-C1-FAK and TirM-TarP-LD. Green cells with >20 adhered bacteria were chosen for live-cell imaging. We found that the majority of FAK recruitment incidences in LD-expressing cells were transient (Fig. 4*E*), consistent with observations from imaging of *Chlamydia* infection and in EPEC-based assays. The transient nature of FAK association made it technically difficult to capture all of the recruitment events in fixed cells, and therefore, values obtained for the steady-state incidence of recruitment were likely to be underestimates.

##### The LD Domain of TarP Bound to FAK in a Manner That Required the Conserved Leucine Residues

The apparent TarP-LD-dependent recruitment of FAK to the plasma membrane strongly suggested interaction. Therefore, we employed the bacterial two-hybrid system to investigate quantitatively the FAK binding of TarP-LD in comparison to that of paxillin LD2 and TarP-mutLD in which the conserved Leu residues were changed to Ala via site-directed mutagenesis. Mutating the conserved leucine residues within paxillin LD2 abrogated FAK interaction (33, 46). In this assay, interaction was reported by the in *trans* reassembly of the active *Bordetella pertussis* adenylate cyclase (Cya) from two constituent fragments (termed 18- and 25-), which are fused to the target and bait proteins, respectively. Target-bait interaction allowed Cya-dependent conversion of ATP to cAMP, reported here as β-galactosidase (β-gal) production. To quantify interaction strength, β-galactosidase-dependent conversion of ONPG to the colorimetric molecule ONP was measured and standardized as Miller units. TarP-LD interaction with FAT was evaluated and directly compared with paxillin LD2 and TarP-mutLD. A positive control Cya18-Zip/Cya25-Zip was included. By measuring β-gal production, it was clear that Cya18-TarP-LD/Cya25-FAT or Cya18-Pax-LD2/Cya25-FAT-co-expressing bacteria exhibited activity that differed significantly from the negative control strain (Fig. 4*F*; *p* < 0.01 ANOVA and Tukey-Kramer post hoc test). Importantly, the Leu-to-Ala substitution mutant did not interact with FAT. Taken together, the data from cell-based assays, structural modeling, and bacterial two-hybrid experiments support a specific and direct interaction between TarP-LD and FAK that required the conserved Leu residues.

Having narrowed down the FAK binding activity, we then addressed the nature of the interaction between FAK and TarP-LD. Structural modeling studies indicated binding could be via hydrophobic interactions between the amphipathic α-helices of TarP-LD and that of FAK FAT domain. The α-helices of the Tarp-LD motifs of *C. trachomatis* and *C. caviae* were superimposed on that of paxillin LD2. A near-perfect coincidence of the hydrophobic residues was observed, including the conserved leucines (data not shown).

**FIGURE 5.**
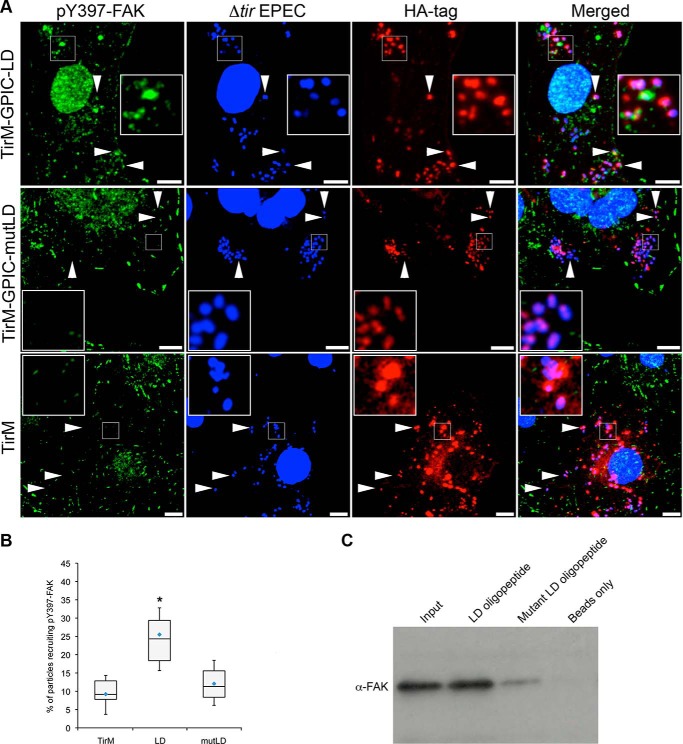
**Recruitment of FAK by TarP-LD domain requires conserved leucine residues.** *A*, Cos7 cells transfected with the indicated plasmid were infected with Δ*tir* EPEC. *Arrowheads* indicate examples of colocalization or lack thereof in the mutant and negative control. Anti-pY397-FAK and anti-HA antibodies were used to visualize pY397-FAK (*green*) or TarP-LD/mutLD (*red*), respectively. Bacteria were visualized by DAPI staining (*blue*). *Insets* show a magnification of a selected area of the cell. *Scale bars*, 10 μm. *B*, adhered EPEC able to recruit pY397-FAK were enumerated, and data are represented as a *box and whisker plot*. Data compiled from three independent experiments. A range of 650–1300 particles was counted. The *asterisk* indicates statistical significance relative to TirM control (ANOVA; *p* < 0.005 and post hoc testing Tukey-Kramer; α = 0.01, q = 4.59). *C*, oligopeptide pulldown using HeLa cell lysates showed that FAK specifically recognizes the TarP-LD motif, whereas amino acid substitutions abrogated interaction. *Lane 1*, 10% input; *lane 2*, wild type LD, *lane 3*, Leu-to-Ala substitution mutant, *lane 4*, streptavidin beads only.

The mutant (TarP-mutLD) was evaluated for the recruitment of pY397-FAK in the EPEC system and the co-precipitation of FAK using custom-synthesized oligopeptides with wild type or mutant LD amino acid sequences. Both experiments were performed in parallel with the wild type TarP-LD and paxillin LD2. As shown in the images in Fig. 5*A* and quantification in Fig. 5*B*, TarP-mutLD exhibited a low level of recruitment similar to the negative TirM control and significantly different to TarP-LD and paxillin LD2. The importance of the leucine residues was further confirmed in a co-precipitation experiment where the N-terminal biotinylated oligopeptide bait containing the Leu-to-Ala-substituted mutant form failed to pull down FAK from whole cell lysates (Fig. 5*C*). Lysates were obtained from cells not treated in any way to induce FAK phosphorylation. Together, the data indicated that, similar to paxillin LD2, the binding of TarP-LD to FAK required the conserved leucine residues.

##### The TarP-LD Domain Recruited the p34^Arc^ Subunit of the Arp2/3 Complex in a FAK-dependent Manner

**FIGURE 6.**
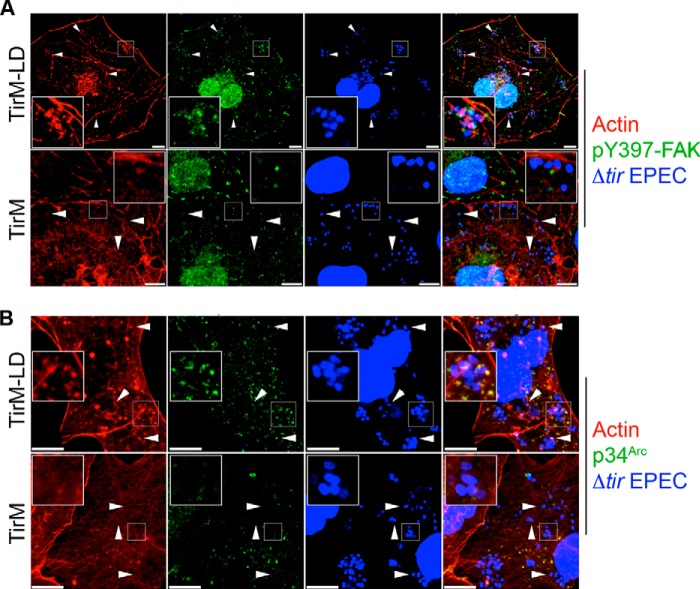
**The TarP-LD domain mediates Arp2/3 complex and actin remodeling.** *A*, Cos7 cells expressing TirM-TarP-LD or the negative control TirM were infected with Δ*tir* EPEC to induce clustering. Transfected cells were identified by their ability to recruit actin (*red*) and pY397-FAK (*green*). The *white arrowheads* indicate colocalization of Δ*tir* EPEC, pY397-FAK, and actin. *B*, as above except p34^Arc^ (*green*) colocalization with bacteria and actin was monitored. *Scale bars*, 10 μm. *Insets* show a magnification of a selected area of the cell.

Using the EPEC system described above, we investigated if TarP-LD could recruit actin and the Arp2/3 complex. Cells expressing TarP-LD or the negative control TirM were infected with Δ*tir* EPEC, and the samples were stained with fluorescent phalloidin to visualize recruited actin. From confocal images, actin assembly in cells expressing TirM-LD was observed, which was in contrast to cells expressing TirM alone (Fig. 6*A*). Furthermore, we observed >75% coincidence of pY397-FAK or p34^Arc^ with actin (Fig. 6, *A* and *B*), supporting a signaling related recruitment of actin.

**FIGURE 7.**
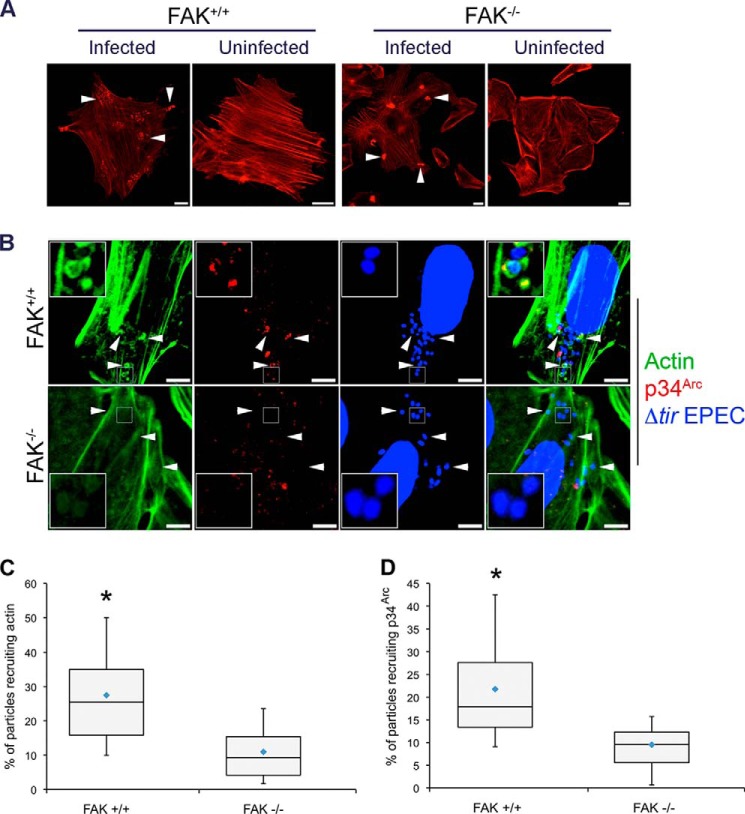
**The TarP-LD domain does not recruit actin or p34^Arc^ in cells lacking FAK.** *A*, FAK^−/−^ cells can support pedestal structures induced by EPEC, indicating that actin recruitment and polymerization (*white arrows*) to the plasma membrane remained functional in the FAK^−/−^ background. *Scale bars*, 10 μm. *B*, FAK^+/+^ and FAK^−/−^ MEFs expressing TirM-LD were infected with Δ*tir* EPEC, and actin remodeling and p34^Arc^ recruitment were monitored. The *white arrowheads* indicate colocalization or lack thereof of actin and p34^Arc^ with Δ*tir* EPEC. *Scale bars*, 10 μm. *C*, colocalization of actin with Δ*tir* EPEC was enumerated, and data are represented as a *box and whisker plot. D*, enumeration of Δ*tir* EPEC colocalization with p34^Arc^ with data represented as a *box and whisker plot*. Data were compiled from three independent experiments. A range of 550–700 particles for each was counted. The *asterisk* indicates statistical significance (ANOVA; Actin and p34^Arc^: *p* < 0.005 and *post hoc* testing Tukey-Kramer; α = 0.01, q = 3.76).

Next, the functional relationship between FAK and the TarP-LD-dependent actin remodeling was investigated in FAK^−/−^ MEFs. Demonstration of FAK-dependent actin remodeling by TarP-LD would support a signaling function for this domain. First, because of the potential actin-related signaling abnormalities the FAK^−/−^ MEFs may have, including the hyper-activation of the RhoA GTPase (47), it was necessary to confirm that these cells remained competent in signaling from the plasma membrane to the actin cytoskeleton. Early-passage FAK^+/+^ and FAK^−/−^ cells, directly obtained from the American Type Culture Collection, were infected with wild type EPEC, and actin-rich pedestal formation was monitored. As shown in Fig. 7*A*, FAK^−/−^ cells supported EPEC pedestal formation, indicating that one or more signaling pathways to induce actin remodeling at the plasma membrane remained intact.

To confirm FAK involvement, FAK^+/+^ and FAK^−/−^ MEFs were transfected with TirM-TarP-LD, and the recruitment of actin and p34^Arc^ was monitored. First, the recruitment of both actin and p34^Arc^ could be observed in the wild type cells (Fig. 7*B*). In contrast, TirM-LD-expressing FAK^−/−^ MEFs were unable to support the recruitment of both actin and p34^Arc^ to the plasma membrane, indicating that FAK was essential for their recruitment. As shown in Fig. 7, *C* and *D*, the recruitments of actin and p34^Arc^ by TirM-LD were largely FAK-dependent, increasing 2.5- and 2.3-fold, respectively, in FAK^+/+^ cells (actin and p34^Arc^: *p* < 0.005, ANOVA and Tukey-Kramer post hoc test). Together, we concluded that TarP-LD signals to FAK to recruit the Arp2/3 complex and actin. This would be consistent with previous published observation demonstrating a requirement for the Arp2/3 complex by *Chlamydia* during invasion (14).

##### Actin Remodeling by TarP-LD Required a Functional Arp2/3 Complex

**FIGURE 8.**
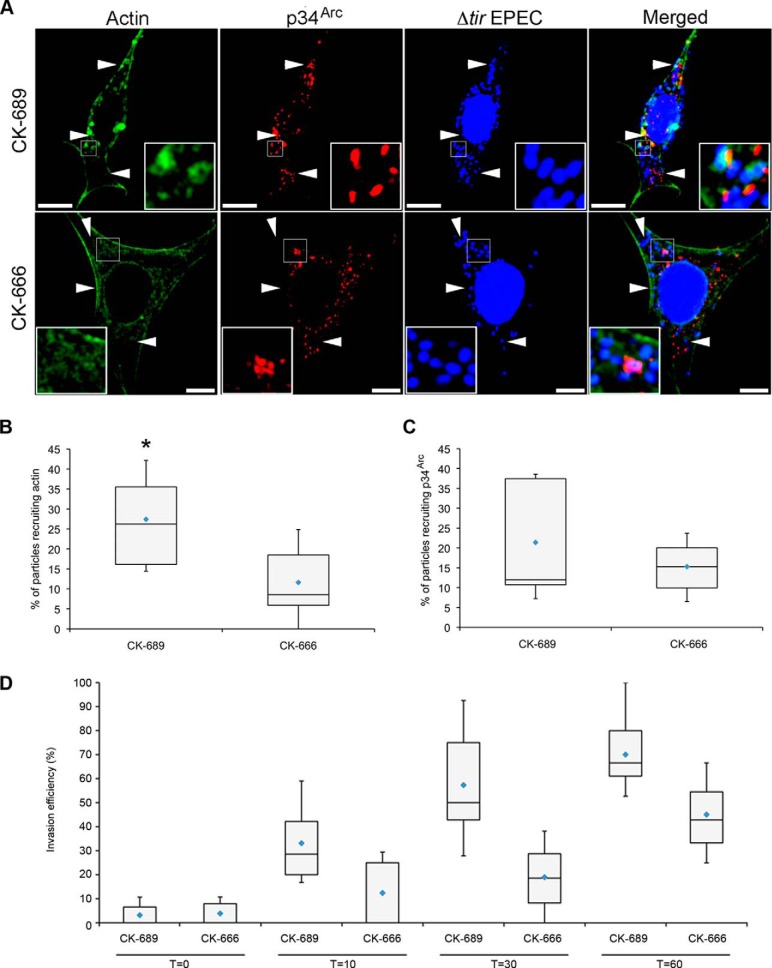
**TarP-LD signaling to actin is dependent on the Arp2/3 activity.** *A*, the LD-mediated recruitment of actin required a functional Arp2/3 complex. FAK^+/+^ MEFs expressing TirM-LD were treated with 100 μm concentrations of the cell-permeable inhibitor CK-666 or the inactive control CK-689 during infection with Δ*tir* EPEC. Actin (*green*) and p34^Arc^ (*red*) were visualized with phalloidin or an anti-p34^Arc^ antibody, respectively. Bacteria were visualized by DAPI. *White arrowheads* indicate colocalization or lack thereof. Note the absence of F-actin aggregates underneath the bacteria on cells exposed to CK-666. The *insets* show a magnification of a selected area of the cell. *Scale bars*, 10 μm. Adhered EPEC colocalizing with actin (*B*) or p34^Arc^ (*C*) were enumerated, and data are presented as box and whisker plots. Data were compiled from three independent experiments. A range of 680–840 particles was counted for each group. The *asterisk* indicates statistical significance (ANOVA; actin, *p* < 0.005; p34^Arc^, *p* < 0.3 and post hoc testing Tukey-Kramer; α = 0.01, q = 3.96). *D*, HeLa cells pretreated with CK-666 or CK-689 were infected with GPIC. Infection was allowed to proceed up to 10-, 30-, and 60-min post-temperature shift. Data were from a minimum of 100 cells from three independent experiments and expressed as a *box and whisker plot* (see “Experimental Procedures” for details on statistical analysis; ANOVA *p* < 0.005 and post hoc testing Tukey-Kramer; α = 0.01, q = 4.7).

To address the potential Arp2/3 requirement, a cell-permeable inhibitor (CK-666) that specifically targeted Arp2/3-mediated actin filament nucleation was used. The inhibitor prevented Arp2/3 from achieving an active conformation by targeting the pocket formed by the subdomain 4 of Arp2 and subdomain 1 of Arp3 without affecting its recruitment to the complex (48). As above, FAK^+/+^ cells were transfected with the TirM-TarP-LD expression constructs. Recruitment of actin and Arp2/3 were visualized with Alexa 488-phalloidin and anti-p34^Arc^, respectively. The cells were pretreated with 100 μm concentrations of either CK-666 or the inactive control, CK-689. In cells treated with the CK-689, recruitment of both actin and p34^Arc^ could be seen. In contrast, CK-666 treatment led to the loss of F-actin aggregates (Fig. 8*A*), indicating that Arp2/3 activation was essential for TarP-LD-mediated actin remodeling. The levels of colocalization of EPEC for either actin or p34^Arc^ were quantified for both CK-666- and CK-689-treated cells. As shown in Fig. 8, *B* and *C*, recruitment of actin, but not p34^Arc^, was significantly reduced in cells treated with CK-666 inhibitor (actin, *p* < 0.005; p34^Arc^, *p* < 0.3, ANOVA, and Tukey-Kramer post hoc test).

Finally, the effects of CK-666 on *Chlamydia* invasion were evaluated to validate the above observations (Fig. 8*D*). The critical role of the Arp2/3 complex has already been shown in cells expressing the C-terminal VCA (WA) domain of N-WASP (14). Here, exposure to 100 μm CK-666 dramatically reduced the efficiency of chlamydial invasion at all time points investigated (*p* < 0.005 ANOVA and Tukey-Kramer post hoc test). The apparent increase in the invasion efficiency at the later time points, regardless of CK-666, was likely due to compensatory pathways activated at later times. Taken together, these results supported a signaling function of the TarP-LD domain to recruit actin in a FAK- and Arp2/3-dependent manner.

##### Cdc42 Functions Downstream of FAK and Upstream of the Arp2/3 Complex to Mediate Actin Remodeling

Subtil *et al.* (20) reported that the small GTPase Cdc42 was recruited at the sites of entry and essential for invasion by *C. caviae* strain GPIC, but the molecular basis of recruitment remains unknown. Cdc42 signals downstream of FAK (45). Therefore, its role in TarP-LD/FAK signaling and its position within the signaling pathway were investigated.

**FIGURE 9.**
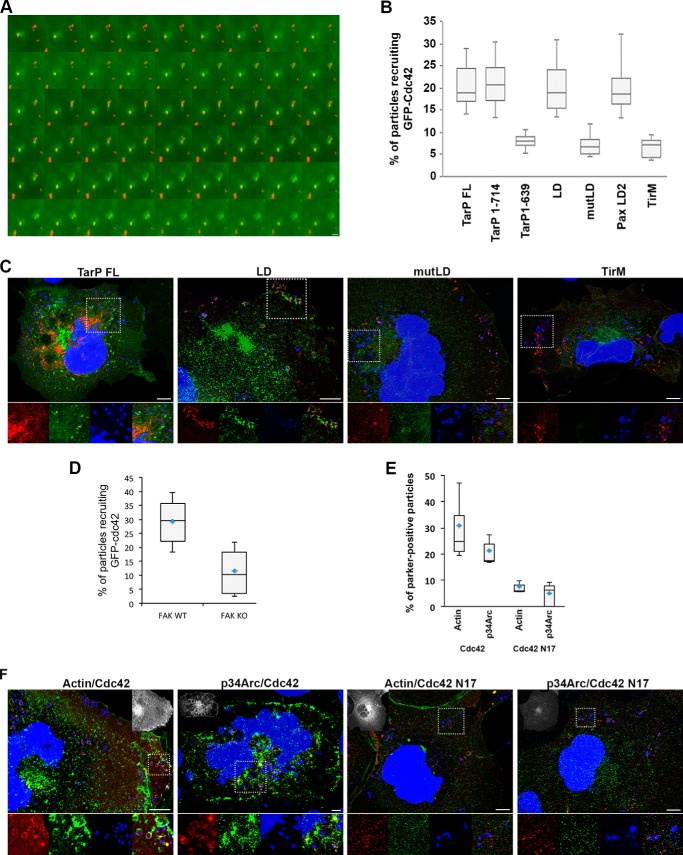
**Cdc42 is important in the TarP-LD- and FAK-dependent recruitment of p34^Arc^ and actin.** *A*, binding of CMTPX-labeled *C. caviae* strain GPIC (*red*) to cells induces a highly localized and transient recruitment of EGFP-Cdc42 (*green*). The images were obtained by live cell microscopy at 30-s intervals. *Scale bar* = 5 μm. *B*, adhered EPEC able to recruit EGFP-Cdc42 were enumerated for the constructs indicated, and data are represented as a *box and whisker plot*. Approximately 500–800 particles were counted for each construct. The *asterisks* indicate significance relative to TirM control (ANOVA; *p* < 0.005 and post hoc testing Tukey-Kramer; α = 0.01, q = 4.80). *C*, representative images analyzed to arrive at the data shown in *B* with a *dotted square border* indicating the region represented in the *inset* images; *Inset* images are of HA-TirM-TarP derivative (*red*), EGFP-Cdc42 (*green*), Δ*tir* EPEC stained with DAPI (*blue*), and merged. *Scale bar*, 10 μm. *D*, EGFP-Cdc42 recruitment requires FAK, as determined using FAK^+/+^ and FAK^−/−^ MEFs. *E*, cells expressing the dominant-negative mutant EGFP-Cdc42N17 showed decreased incidence of actin remodeling and p34^Arc^ recruitment at the sites of bacterial adherence. *F*, representative images analyzed to arrive at the data shown in *E* with a *dotted square border* indicating the region represented in the *inset* images; *inset* images are of HA-TirM-TarP derivative (*red*), actin or p34Arc (*green*), Δ*tir* EPEC stained with DAPI (*blue*), and merged. An additional inset image (*grayscale*) is included to demonstrate expression of either EGFP-Cdc42 or EGFP-Cdc42N17 mutant. *Scale bar*, 10 μm.

First, the recruitment of Cdc42 reported by Subtil *et al.* (20) was verified by infecting EGFP-Cdc42-expressing cells with CMTPX-labeled *C. caviae* strain GPIC and monitoring EGFP-Cdc42 recruitment by live-cell microscopy (Fig. 9*A*) at 30-s intervals. As with EGFP-FAK, the recruitment was rapid and transient. Having established Cdc42 recruitment during chlamydial invasion, the molecular basis for this event was investigated using the EPEC system described above. Different deletion derivatives of TarP were evaluated for their ability to support the recruitment of EGFP-Cdc42 to the plasma membrane. By enumerating the incidence of recruitment, we found that those constructs that retained recruitment of FAK and actin also were able to recruit Cdc42. Fig. 9*B* showed that TarP^1–714^ was able to support Cdc42 recruitment, but progressive deletion (TarP^1–639^) significantly reduced recruitment incidence to the level of the negative control TirM (3-fold difference *versus* TirM; *p* < 0.005). Furthermore, TarP-LD was sufficient to recruit Cdc42 (3-fold difference *versus* TirM; *p* < 0.001). Importantly, the conserved Leu residues were essential, with TarP-mutLD displaying a 3-fold decrease in EGFP-Cdc42 recruitment (Fig. 9*B*). Fig. 9*C* shows representative images of EGFP-Cdc42 recruitment by select TirM-TarP constructs.

To further establish the possible role of Cdc42 within the TarP-LD/FAK pathway, Cdc42 recruitment by TarP-LD was monitored in FAK^−/−^ and FAK^+/+^ MEFs. Fig. 9*D* showed an ∼3-fold difference in Cdc42 recruitment frequency in the WT and KO cells, placing Cdc42 downstream of FAK in the TarP-LD signaling pathway.

The role of Cdc42 in actin remodeling and p34^Arc^ recruitment was also investigated through the use of the dominant-negative mutant (Cdc42N17) (40). Cells were co-transfected with EGFP-Cdc42N17 and the various TirM-TarP constructs. They were infected with Δ*tir* EPEC, fixed, and processed for immunofluorescence staining and microscopy. Total adhered bacteria and those recruiting p34^Arc^ were enumerated. As shown in Fig. 9*E*, the expression of the dominant negative mutant of Cdc42 significantly reduced p34^Arc^ recruitment and actin remodeling at the sites of EPEC adhesion. The incidence of EPEC-localized polymerized actin was reduced by 6-fold in EGFP-Cdc42N17-expressing cells. Consistent with this was the ∼4-fold reduction in p34^Arc^ recruitment in cells expressing the Cdc42N17 mutant (Fig. 9*E*).

Together, the data are consistent with the findings of Subtil *et al.* (20) that Cdc42 is important for actin remodeling during invasion by *C. caviae* serovar GPIC. Importantly, our data also provided a molecular basis for Cdc42 recruitment by placing this GTPase within the context of TarP signaling.

## DISCUSSION

The FAK has been implicated in *Chlamydia* invasion, with Coombes and Mahony (21) first reporting its phospho-activation during infection by *C. pneumoniae*. Subsequently, an extensive RNA interference screen identified FAK and other focal adhesion-associated proteins as important in *C. trachomatis* infection of cultured cells (27, 28). In this report we confirmed that FAK was indeed important for *Chlamydia* invasion and provided a molecular mechanism for its recruitment to the sites of entry. Through deletion analysis of TarP, the mutagenesis of the putative FAK binding site in TarP (TarP-LD) and the elimination of the binding partner FAK through the use of FAK^−/−^ MEFs, we were able to conclude that FAK recruitment at the sites of entry was due to the recognition of a paxillin LD2-like motif in TarP by FAK and that this interaction was important for the recruitment of actin in a Cdc42- and Arp2/3-dependent manner.

Our data also further clarified the involvement of Cdc42 in actin assembly and invasion by *C. caviae* by providing a molecular basis for its recruitment. In addition, our findings also placed Cdc42 within the TarP signaling pathway, having an important role in the recruitment of the Arp2/3 complex.

The Arp2/3 complex can bind to the FERM domain of FAK, and this binding is influenced by the phosphorylation state of FAK at residue Tyr-397 (49). Although this may also function in *Chlamydia* invasion, the Cdc42-dependent Arp2/3 recruitment may be the preferred pathway based on two critical pieces of data. First, the recruitment of the Arp2/3 complex was dependent on Cdc42. Second, there was a robust phosphorylation of FAK at Tyr-397, and this modification was reported to inhibit Arp2/3 complex interaction with the FERM domain of FAK (50).

Direct binding of Cdc42 to FAK has not been reported (51) but may nonetheless function within a signaling complex. Therefore, how Cdc42 is activated in this pathway would likely involve the recruitment of negative and positive regulators of Cdc42. One possibility is the DOCK family of proteins, which was recently demonstrated to activate both Rac and Cdc42 (52). The RNA interference screen conducted by Elwell *et al.* (27) identified DOCK180 as essential in *Chlamydia* infection. It remains to be seen if Rac also functions in the TarP-LD/FAK signaling pathway. This report provides the framework for further detailed studies.

The findings of a signaling function for the LD motif of TarP is in contrast to the findings by Jiwani *et al.* (18), who showed an F-actin binding/bundling activity of TarP via the newly discovered FAB1 and FAB2 domains, the former overlapping with the LD domain. The differences in results could be explained by the nature of the experimental systems with F-actin binding demonstrated primarily using *in vitro* assays, whereas the FAK interaction and signaling functions were identified in a cell-based experimental system. They also demonstrated F-actin localization to TarP aggregates that formed upon overexpression (18). We conducted a similar experiment and found that in addition to F-actin, FAK also associated with the TarP aggregates (data not shown). Refining this experimental system by shortening the transfection time to 6–8 h revealed the preferential colocalization of TarP-LD with FAK in focal adhesions.

An important aspect of our EPEC/TirM-based experimental model was that it enabled the functional evaluation of TarP at the plasma membrane, where it functions during infection. In this context we observed that actin remodeling was dependent on an intact LD domain, FAK, Cdc42, and the Arp2/3 complex. These observations favor a signaling function for the TarP-LD, which is consistent with previous findings with the phosphodomain of the *C. trachomatis* TarP orthologue and with recent observations of TarP-vinculin association.^6^ Furthermore, mathematical modeling based on quantitative imaging data also indicated that the respective signaling pathways of FAK and vinculin exhibited functional interactions,^6^ reinforcing our hypothesis that the TarP-LD motif has a signaling function. We would like to emphasize that we do not exclude an F-actin binding function for this domain, as it may well be relevant to post-invasion stages, specially with *Chlamydia* seemingly capable of manipulating the host cell cytoskeleton throughout its infection (51).

In summary, we present evidence for a signaling function of the TarP-LD domain through its interaction with FAK in a similar binding strategy as its native binding partner, paxillin. The emerging modular architecture of TarP highlights a multifunctional potential, possibly through the combined actions of the different domains. Furthermore, TarP may also display functional cooperation with host receptors previously implicated in *Chlamydia* invasion (27, 52) to ensure efficient invasion, thus benefiting the pathogen. A better understanding of the full extent of TarP function and its regulation should contribute to the breadth of our knowledge of the subversive strategies employed by bacterial pathogens in addition to revealing unique aspects of *Chlamydia*-host interactions.

## Footnotes

5 R. A. Carabeo, unpublished data.

6 T. R. Thwaites, A. T. Pedrosa, T. Peacock, A. T. Nogueira, M. Thiel, and R. A. Carabeo, manuscript in preparation.

## Footnotes

4 The abbreviations used are:

FAB1: F-actin binding domain 1
FAK: focal adhesion kinase
FAT: focal adhesion-targeting
EPEC: enteropathogenic *E. coli*
GPIC: guinea pig inclusion conjunctivitis
FL: full-length
p.i.: post-infection
CMTPX: 4-({[4-(chloromethyl)phenyl]carbonyl}amino)-2-(1,2,2,4,8,10,10,11-octamethyl-10,11-dihydro-2H-pyrano[3,2-g:5,6-g′]diquinolin-1-ium-6-yl)benzoate
Tir: translocated intimin receptor
MEF: mouse embryo fibroblast
pY397-FAK: Tyr^397^-autophosphoryled FAK
ANOVA: analysis of variance
EGFP: enhanced GFP
EB: elementary body
LD: leucine-aspartic acid.
